# Transient impact of chronic social stress on effort-based reward motivation in non-food restricted mice: Involvement of corticosterone

**DOI:** 10.1016/j.ynstr.2024.100690

**Published:** 2024-11-09

**Authors:** Danina Evertse, Pilar Alves-Martinez, Giulia Treccani, Marianne B. Müller, Frank J. Meye, Michael A. van der Kooij

**Affiliations:** a Department for Developmental Origins of Disease, Wilhelmina Children's Hospital, Utrecht University, Utrecht, the Netherlands; b Leibniz Institute for Resilience Research (LIR), Mainz, Germany; c Department for Systemic Neuroscience, Institute of Anatomy and Cell Biology, University of Marburg, Marburg, Germany; d Department of Psychiatry and Psychotherapy, Translational Psychiatry, University Medical Center of the Johannes Gutenberg University, Mainz, Germany; e Department of Translational Neuroscience, Brain Center, UMC Utrecht, Utrecht University, Utrecht, the Netherlands

**Keywords:** *Social stress*, *Corticosterone*, *Mice*, *Motivation*

## Abstract

Chronic stress has been connected to a reduced effort and motivational deficits. To study effort-based motivation in rodents, operant conditioning is often employed. However, caloric restriction is typically imposed simultaneously. Since caloric restriction is a stressor in its own right, this procedure interferes with data interpretation. Here, we investigate whether chronic social defeat stress (CSD), lasting 10 consecutive days, would alter effort-based reward motivation in mice trained under *ad libitum* food conditions. Utilizing operant FED3 boxes in home cages, mice were trained within eight days to nose poke for palatable food. After training completion, operant memory was retained for at least 16 days, and mice demonstrated sustained effort, as assessed with a progressive ratio schedule, to obtain reward pellets. Directly after CSD exposure (10th day), mice exhibited reduced effort for palatable food rewards, but also displayed reduced nose poking in general. The effects of CSD on effort were short-lived, with no lasting impact on effort-based reward motivation one week post-stress. As corticosterone (CORT) levels were increased at day 10 of CSD, but not at day 17, we hypothesized that CORT might mediate the acute effects of CSD on effort-based reward motivation. Indeed, CORT administration [100 μg/ml], supplied via the drinking water, mirrored the CSD-induced CORT spike and temporarily reduced reward motivation. Our findings emphasize that CSD does not result in long-term deficits in reward motivation, suggesting a resilient adaptive response in mice under unrestricted feeding conditions. This study underscores the necessity of considering temporal dynamics of stress impacts and highlights the modulating effects of CORT. These insights contribute to a deeper understanding of the resilience mechanisms in motivational impairments and pave the way for further research into factors facilitating this resilience.

## Highlights

• Without food restriction, mice are fully trained on operant FED3 in 8 days.
• Acute impact social stress on reward effort is linked to hypercortisolemia.
• Stressed mice maintained long-term reward motivation, suggesting stress resilience.

## 1 Introduction

Stressful life events are strongly implicated in the etiology of mood disorders such as major depression (Kendler et al., 1999; Pittenger and Duman, 2008). Notably, a meta-analysis identified an association between effort-reward imbalances and risk for depression (Rugulies et al., 2017). Impairments in effort, or motivational deficits (sometimes referred to as apathy or avolition), are a central element in the pathology of depression (Husain and Roiser, 2018; Salamone and Correa, 2024). To study effort-based reward motivation in greater detail, animal models are employed (Gould and Gottesman, 2006). Frequently employed animal models to study the impact of chronic stress include chronic restraint stress, chronic unpredictable stress and chronic social defeat (van der Kooij, 2020). In agreement with human literature, stress exposure to rodents reduces effort-based reward motivation during operant conditioning (Cryan and Slattery, 2017). Of note, however, social defeat in rats has also been reported to enhance effort-based reward motivation (Riga et al., 2015).

To promote task engagement during operant conditioning, animals are typically food deprived, whereby bodyweights are kept at 85–90% of their normal weight (Brady and Floresco, 2015; Mar et al., 2013). Food deprivation in mice affects the animal's hormonal balance, glucose metabolism and behavior (Jensen et al., 2013; Peña-Villalobos et al., 2020). Food deprivation becomes particularly problematic when studying the effects of chronic stress, since food restriction enhances stress responses and affects reward-related -as well as other-behaviors (Beck and Luine, 1999; Stamp et al., 2008). For example, food deprived mice display a heightened release of corticosterone (Mantella et al., 2005), but chronic corticosterone administration to mice has also been found to impair positive valence behaviors associated with reward processing (Peng et al., 2021; Dieterich et al., 2019).

A further complication stems from the divergent impact of caloric restriction between stressed animals and controls, seeing that social stress can result in a -temporary- negative energy balance (Coccurello et al., 2018; Jene et al., 2021; Linders et al., 2022). Hence, to study the effects of stress on reward motivation, food restriction comprises an important confounding factor that ought to be avoided, echoing the earlier critiques voiced by Harry Harlow (1953). Yet, published studies investigating the effects of chronic stress on effort-based reward motivation have imposed (mild) caloric restriction to the animals tested (Bergamini et al., 2016; Bergamini et al., 2018; Kúkel'ová et al., 2018; Dieterich et al., 2021; Yoshida et al., 2021). We therefore question whether the claim, stating that chronic stress impairs effort-based reward motivation, can be maintained without food deprivation.

Second, the effects of stress on effort-based reward motivation are often assessed in close temporal proximity to chronic social defeat (CSD); i.e. overlapping with the days on which social defeat also occurred) (Bergamini et al. 2016, 2018; Yoshida et al., 2021). However, it has repeatedly been shown that CSD impairs motor activity for several days after stress (McGaughey et al., 2019; Ota et al., 2018; Zhu et al., 2022). Thus, a stress-induced reduction in general activity during operant conditioning may lead to an overall lower response rate in stressed animals, as opposed to a specific reduction in reinforced responses. Of note, there is important overlap between motor control and motivation and it may, thus, not always be feasible to disentangle these two processes (Salamone and Correa, 2024). Seeing that depressed patients exhibit reduced locomotor activity (Todder et al., 2009), hypoactivity may be an inherent aspect of depression.

Finally, another limitation of classical operant conditioning is that typically effort-based food intake is only monitored for about 1–2 h per day, which precludes investigating the impact of the circadian rhythm. It would thus be an of added value to increase the duration of observation to fully take into account the potential effects of chronic stress on effort-based reward motivation.

**Fig. 1:**
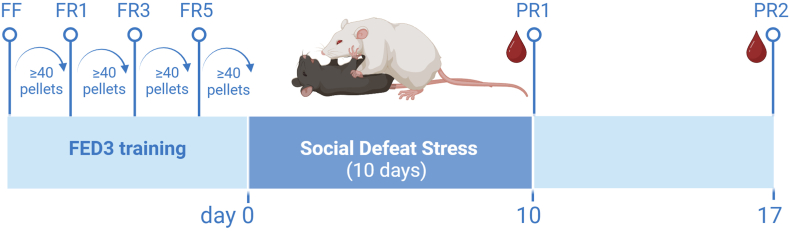
Experimental overview of the study. First, mice were trained for operant conditioning on the FED3 boxes during four phases. Mice proceeded to the next phase if at least 40 reward pellets were obtained. Phase progression: free feeding (FF) and fixed ratio (FR)1, FR3 and FR5. Thereafter, mice were subjected to chronic social defeat stress, lasting 10 consecutive days. Mice were assessed for effort-based reward motivation, based on a progressive ratio (PR), on day 10 (PR1) and one week thereafter (PR2).

The recently developed Feeding Experimentation Device version 3 (FED3) (Matikainen-Ankney et al., 2021) can be considered as an alternative to classical operant conditioning, which allows mice to be trained 24/7, at their own pace, and tested in their stress-free home environment. Using the FED3 systems, we demonstrate that mice are fully trained in about a week to repeatedly nose poke for a palatable food reward, with *ad libitum* food (chow) availability. Under these non-restricted conditions, we assessed whether CSD stress altered effort-based reward motivation. Since timing is an important consideration in evaluating the effects of social stress on behavior (Jene et al., 2018), we investigated the effects of CSD on effort-based reward motivation in the FED3 immediately after conclusion of the stressor as well as one week thereafter (Fig. 1).

## 2 Methods

### 2.1 Animals

Adult male C57BL/6 J mice (Janvier, France) arrived at the animal facility at 8–10 weeks of age and were habituated to the housing conditioning (T: 23 ± 2 °C, humidity: 50 ± 5 %) for at least 1 week before experiments commenced. Mice were single-housed on a 12/12 h light-dark cycle (lights on 7 a.m., lights off 7 p.m.). Chow pellets (on top of the grid) and water were available *ad libitum* throughout the experiment. C57BL/6 J mice were randomly assigned as controls or to the treatment group (CSD, corticosterone). Adult male CD-1 retired breeders (Janvier, France), were used as aggressors in the CSD paradigm and were trained/preselected based on their tendency for aggressive behavior (latency to attack <60s) (Golden et al., 2011). Mice were terminated by i.p. Injection of pentobarbital (150 mg/kg b.w.). All experiments were performed in accordance with the European Directive 2010/63/EU for animal experiments. Experiments involving CSD were approved by the local authorities in Germany (license G20-17-058, Animal Protection Committee of the State Government, Untersuchungen Rheinland-Pfalz, Koblenz). Experiments involving corticosterone treatment were approved by the Animal Ethics Committee of Utrecht University and the Dutch Central Authority for Scientific Procedures on Animals (CCD), and were conducted in agreement with the Dutch law (Wet op de Dierproeven, 2014).

### 2.2 Operant behavior: training to nose poke for reward

Mice were individually trained for operant behavior using the Feeding Experimental Devices version 3.1 (FED3, Open Ephys, https://hackaday.io/project/106885-feeding-experimentation-device-3-fed3). The FED3 box is a smart pellet dispenser to train mice on operant tasks which we attached to the home cage (Matikainen-Ankney et al., 2021). The FED3 box is equipped with two nose poke holes and a pellet dispenser. The FED3 box detects when the mouse pokes in the holes and allow food rewards to be dispensed, as directed per program. The code used for the programs is all open source (GitHub – KravitzLabDevices/FED3); we used ClassicFED3 Program. The FED3 boxes were equipped with purified dustless precision pellets weighing 20 mg each (F0071; PLEXX, The Netherlands), serving as reward, owing to their slightly higher sucrose content. Completion of the training protocol (below) takes approximately one week.

FED3 training: Mice were first habituated for 24 h to the (switched off) FED3 box and the pellets; the FED3 box was attached to the home cage with several pellets being placed in the cage bedding, close to the FED3 box and one pellet in the pellet dispenser. The following day, the FED3 was switched on. From thereon, we daily checked for pellet retrieval and if the mouse obtained ≥40 cumulative pellets on a given phase, it would proceed to the next one (FreeFeeding [FF] - > FixedRatio 1 [FR1] - > [FR3] - > [FR5]). FF: a pellet gets dispensed as soon as the last pellet is taken out of the dispenser and no nose pokes are required to obtain a pellet. FR1: mice are required to poke the correct (active) hole once to receive a pellet. The incorrect hole (inactive) served to control for pellet-dissociated responses. During the following FR3 and FR5, three and five nose pokes in the correct hole were respectively required to obtain a food pellet. When the mouse obtained ≥40 pellets during FR5, it was considered to be fully trained. After training, each cohort of mice was split into two equal groups, based on their FED3 performance (# correct nosepokes) at the last training day on FR5, such that pre-treatment training performance was similar. Thereafter, these groups were randomly assigned to their respective treatment or control condition.

### 2.3 Chronic social defeat paradigm

During CSD, male C57BL/6 J mice were defeated three times/day between 10 a.m. and 2 pm for 10 consecutive days. The C57BL/6 J mice were introduced to the home cage of different unknown male CD-1 aggressor mice and subsequently defeated; these social encounters ended after a cumulative 10 s of CD-1 aggression. In between aggressive encounters, animals were separated for 15 min through a perforated partition to avoid further physical contact while allowing other sensory contact, such as smell and visual and auditory cues. Following the three daily defeats on a given day, intruder and aggressor were co-housed in the same cage but were physically separated using the partition until the following day. Control mice (CTRL) were exposed to a novel clean cage (90 s) for 10 consecutive days and their home cages were also equipped with a perforated partition, and a cage mate behind this partition, to closely mimic the environmental aspects of CSD.

### 2.4 Corticosterone treatment

For 10 consecutive days, mimicking CSD duration, animals were treated with either a vehicle (0.45% hydroxypropyl-β-cyclodextrin, Cat#332593, Sigma-Aldrich) or with CORT (35 μg/ml [low dose] or 100 μg/ml [high dose], Cat#C2505, Sigma-Aldrich, dissolved in vehicle), in their drinking water (Agasse et al., 2020). Since CORT is light sensitive, the liquid treatments were administered via opaque drinking bottles. The solutions in the drinking bottles were replenished after five days.

### 2.5 Operant behavior: effort-based reward motivation (progressive ratio, PR)

After stress-exposure (CSD, CORT) or respective control-treatment, mice were allowed access to the FED3 again and assessed for effort-based reward motivation using a Progressive Ratio (PR). The PR lasted 24 h and was performed acutely (day 10) and/or at long-term (day 17). During PR, the nose pokes required to receive a pellet increased exponentially, using Euler's e, following $PokesRequired=\left[5e^{PelletCount*0.2}\right]-5$ (Richardson and Roberts, 1996), rounded to the nearest integer. Reward motivation was estimated by the number of pokes in the active port and the number of reward pellets obtained.

### 2.6 Blood sampling and corticosterone measurements

Mice were placed on a grid and gently held by the tail after which a nick was made to the lateral tail vein. The first drop of blood was discarded after which 40 μl of blood was collected in an EDTA-containing tube, as published (van der Kooij et al., 2018). To facilitate hemostasis, a tissue was pressed against the wound for 5–10 s. During the blood sampling procedure, taking place between 1 and 3 pm, mice could freely move. Blood sampling was about 1 h apart from other interventions (e.g. commencement of behavioral testing, induction of stress). Of note, blood sampling by tail nick did not affect the levels of circulating corticosterone (Fluttert et al., 2000; Jene et al., 2018). Collected blood was centrifuged for 10 min at 10,000 rpm at 4 °C. The resulting supernatant (plasma) was then collected and stored at −80 °C. With a CORT ELISA kit (Cat#ADI-900-0979, Enzo Life Sciences) the concentrations of CORT in the plasma samples were measured, according to the manufacturer's instructions.

### 2.7 Statistics

All data and graphs were analyzed and made using Prism version 6 (GraphPad Software, Inc.). Statistical outliers were defined as values ± three times S.D. from the mean and excluded from further analysis. We also checked whether our data was normally distributed and adjusted accordingly. Unpaired two-tailed student's *t*-test, or their non-parametric alternative, was used to compare data between CSD and CTRL. For the CORT response in blood plasma a two way ANOVA with repeated measurements was used and, after finding a main effect, followed up with a Šídák's multiple comparisons post-test. Seeing that CSD inhibited effort-based reward motivation on day 10, we used an unpaired one-tailed student's t-test to compare outcome between CORT and VEH-treated mice in our CORT experiments [100 μg/ml], where we mimicked the CORT response of CSD on day 10.

## 3 Results

### 3.1 Training for operant conditioning in mice is swift and accurate

**Fig. 2:**
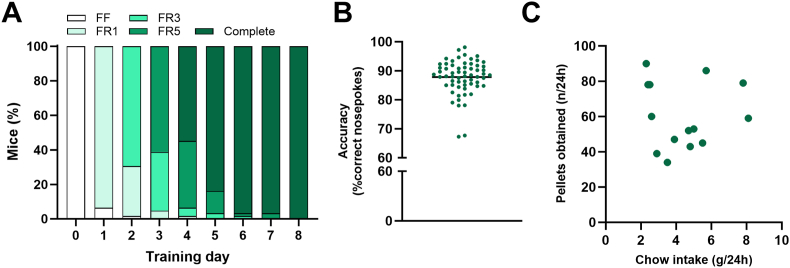
Progression of mouse training on FED3 boxes. (**A**) Mice completed training within 8 days, by obtaining ≥40 reward pellets during FR5. (**B**) All mice displayed high levels of accuracy (% correct nosepokes) upon of training completion (87.8% ± 0.7, n = 62). (**C**) Chow intake did not correlate with the number of reward pellets obtained (r^2^ = 0, *P* = 0.96, n = 14). Abbreviations, FF: free feeding, FR: fixed ratio. Statistical test used is Pearson correlation (**C**).

Mice were fully trained for operant conditioning using the FED3 boxes attached their home cage in 4.7 ± 0.1 d (Fig. 2A), by obtaining a minimum of 40 reward pellets at the FR5 phase. Fully trained mice displayed an accuracy of 87.8 ± 0.7 % at the end of the final (FR5) training phase (Fig. 2B). Chow intake during FR5 (taking the final 24 h) did not correlate with the number of reward pellets obtained in this time span (Fig. 2C), suggesting that the reward pellets did not simply serve as an alternative food source to standard chow.

### 3.2 CSD transiently impairs effort based reward motivation, but also affects incorrect responses

**Fig. 3:**
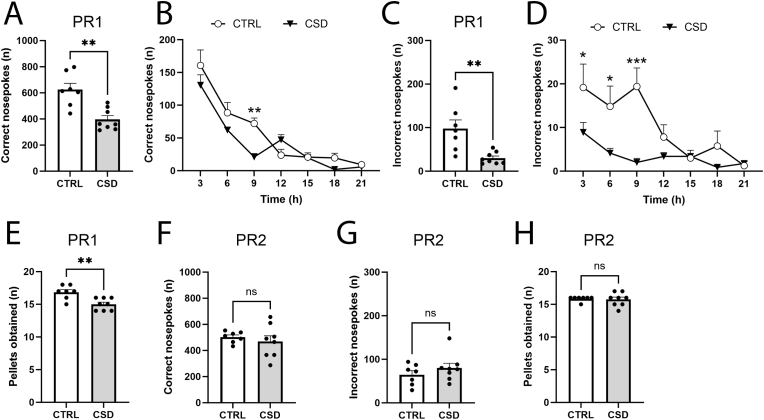
CSD acutely impacted effort-based reward motivation but no effects were found at long-term. (**A**) At PR1 (day 10), CSD-exposed mice displayed decreased number of correct nosepokes (t = 4.15, df = 13, *P* = 0.001, n = 7–8/group). (**B**) Plotting the PR1 correct nosepokes data in 3 h time bins shows that the difference between CTRL and CSD pertains to the first 9 h of the PR (Effect of stress: F_1,77_ = 7.54, *P* = 0.008, 9 h: t = 3.51, *P* = 0.005). The first 3 h took place in the light-phase, followed by 12 h of dark phase (time points 6–15). (**C**) CSD-exposed mice displayed lower number of incorrect nosepokes (t = 3.5, df = 13, *P* = 0.004, n = 7–8/group). (**D**) 3 h time bin data during PR1 reveals that the lowered number of incorrect nosepokes seen for CSD mice concerns the first 9 h of the PR (Effect of stress: F_1,77_ = 26.26, *P* < 0.001, 3 h: t = 3.17, *P* = 0.02; 6 h: t = 3.13, *P* = 0.01; 9 h: t = 5.08, *P* < 0.001). The first 3 h took place in the light-phase, followed by 12 h of dark phase (time points 6–15). (**E**) Stress-exposed mice obtained less reward pellets in comparison to non-stressed controls (t = 3.61, df = 13, *P* = 0.003, n = 7–8/group). One week thereafter (PR2), CSD-exposed mice did not differ in the number of (**F**) correct nosepokes (t = 0.69, df = 13, *P* = 0.50, n = 7–8/group), (**G**) incorrect nosepokes (t = 1.03, df = 13, *P* = 0.32, n = 7–8/group) or (**H**) obtained reward pellets versus CTRL (t = 0, df = 14, *P* = 1.0, n = 7–8/group). PR: progressive ratio. ∗*P* < 0.05, ∗∗*P* < 0.01. Abbreviation, PR: progressive ratio. Statistical tests used are two-way ANOVA (**B**,**D**) and Student's t-test (**A**,**C**,**E**-**H**).

Fully trained mice subjected to CSD or treated as unstressed controls were assessed for effort based reward motivation in progressive ratio (PR) tests. First, we assessed the acute effects of our CSD-procedure, by performing PR1 on the final (10th) day of CSD. We observed that stressed mice exhibited lower correct and incorrect nosepokes (Fig. 3A–D), which were particularly noticeable during the first 9 h of the PR, and mice also obtained fewer reward pellets (Fig. 3E). To investigate whether the effects of CSD on reward motivation would translate into long-term effects, PR2 was performed one week post-CSD (i.e. day 17). During PR2, the number of correct nosepokes (Fig. 3F), incorrect nosepokes (Fig. 3G) and the amount of food pellets obtained did not differ between CSD and CTRL (Fig. 3H), suggesting that the effects of CSD on effort-based reward motivation may be transient only.

### 3.3 Despite the stressful impact, CSD does not affect long-term reward motivation

**Fig. 4:**
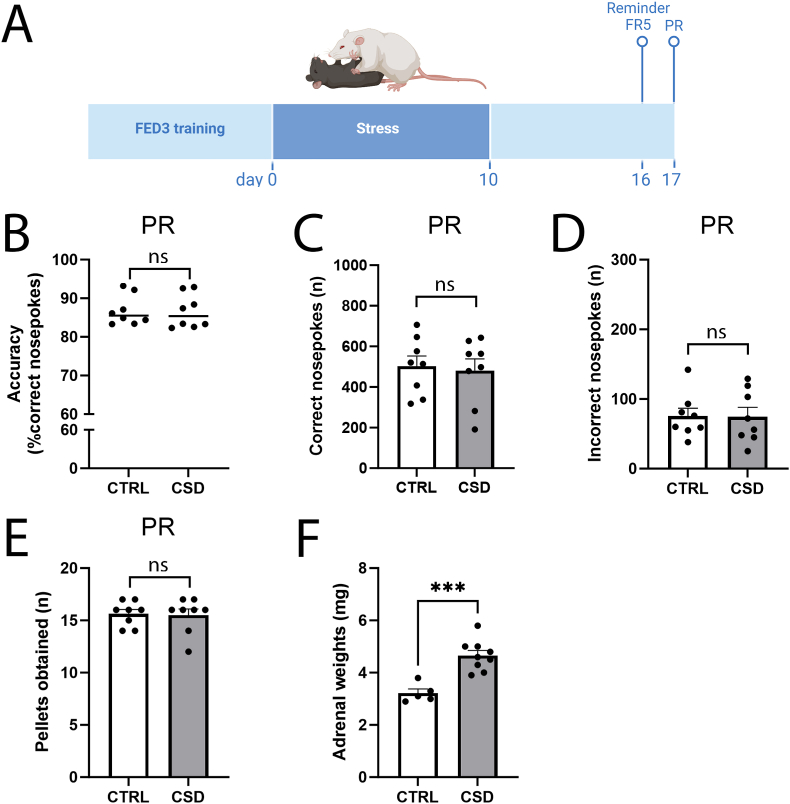
Chronic social defeat stress does not impact effort-based reward motivation one week post-stress. (**A**) Another cohort of mice received FED3 training, were exposed to the CSD paradigm (10 consecutive days of stress) and assessed for training memory (FR5, data in Supplementary Fig. 1) on day 16, and for effort-based reward motivation (PR) at day 17. (**B**) Accuracy during PR is not different between CTRL and CSD mice (t = 0.1, df = 14, *P* = 0.92, n = 8/group). (**C**) The frequency of correct nosepokes did not differ between CTRL and CSD mice (t = 0.29, df = 14, *P* = 0.77, n = 8/group) and (**D**) we also found no difference for the number of incorrect nosepokes (t = 0.05, df = 14, *P* = 0.96, n = 8/group). (**E**) The number of reward pellets obtained during PR did not differ between CTRL and CSD mice (U = 31, *P* = 0.96, n = 8/group). (**F**) The stressful impact of our CSD paradigm was demonstrated by the increased adrenal weights, found three weeks post-CSD (t = 4.96, df = 12, *P* = 0.0003, n = 5 for CTRL and n = 9 for CSD). ∗∗∗*P* < 0.001. Abbreviations FR: fixed ratio, PR: progressive ratio. Statistical tests used are Student's t-test (**B**-**D**, **F**) and Mann-Whitney test (**E**).

To investigate the impact of CSD on long-term reward motivation, without the potential additional effects of performing multiple PRs, another cohort of mice was trained and tested during one PR only, performed 7 d post-CSD (Fig. 4A). Seeing that the delay between training and PR testing now lasted 17 d, mice were first assessed on a reminder trial (FR5), at 6 d post-CSD, to ascertain if the animals’ memory for operant conditioning was retained. During this reminder trial, mice displayed high accuracy. The number of correct and incorrect nosepokes, as well as the number of pellets obtained did not differ between treatment groups (Supplementary Figs. 1A–D). The following day, during PR (7 d post-CSD), accuracy, number of correct nosepokes, number of incorrect nosepokes and the reward pellets obtained were not different between CSD and CTRL (Fig. 4B–E). The stressful nature of our CSD paradigm was demonstrated by enlarged adrenals found three weeks after stress (Fig. 4F), hinting at HPA axis activation (van der Kooij et al., 2018; Jene et al., 2021).

### 3.4 CORT is increased acutely after chronic social defeat stress and CORT-supplementation to the drinking water replicates the short-term effects of CSD on effort-based reward motivation

**Fig. 5:**
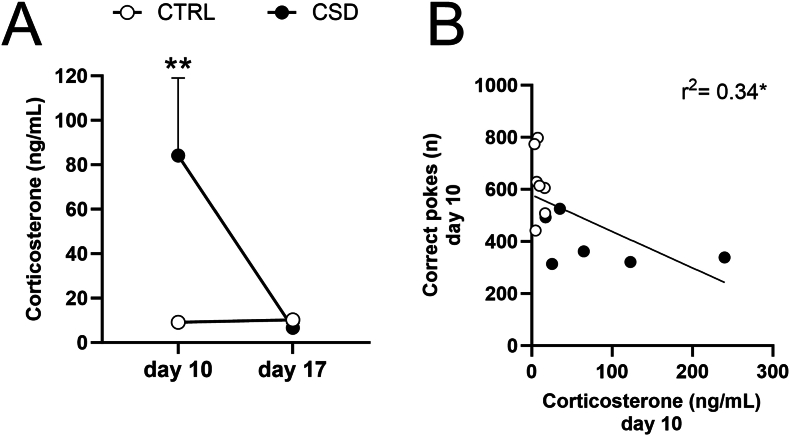
Corticosterone is increased after chronic social defeat stress and correlates negatively with the number of correct nosepokes. (**A**) Plasma corticosterone levels were increased for CSD-exposed mice on day 10, but not at day 17 (Effect of stress: F_1,14_ = 5.06, *P* = 0.04; day 10: t = 3.36, *P* = 0.005; day 17: t = 0.14, *P* = 0.99, n = 6–7/group). (**B**) Corticosterone levels at day 10 correlated negatively with the number of correct nosepokes displayed during the progressive ratio performed on day 10 (r^2^ = 0.34, *P* = 0.039, n = 13). ∗*P* < 0.05, ∗∗*P* < 0.01. Statistical tests used are two-way ANOVA (**A**) and Pearson correlation (**B**).

**Fig. 6:**
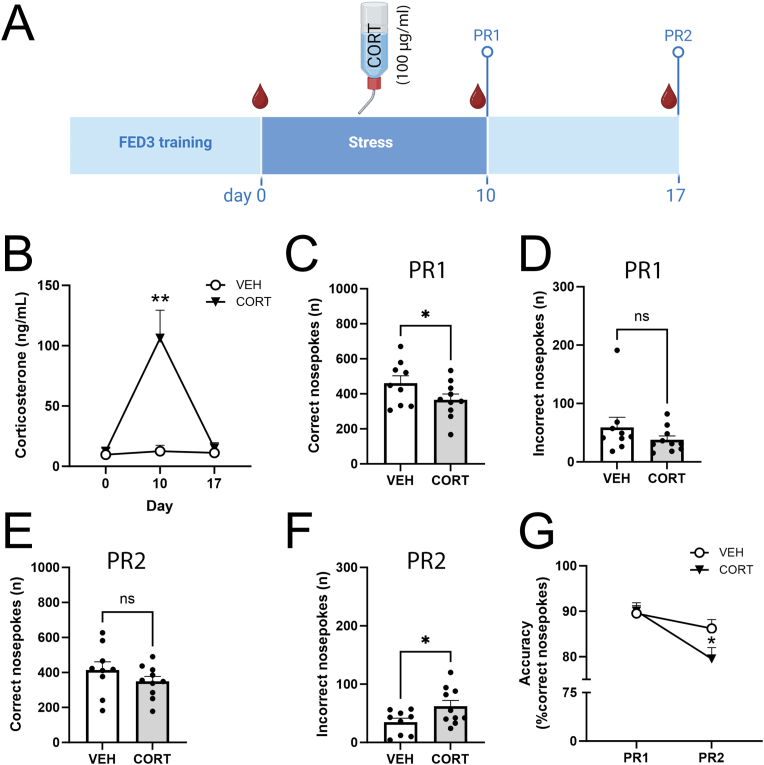
Corticosterone administration [100 μg/ml] via the drinking water mimics the physiological impact of chronic social defeat and the behavioral effect on effort-based reward motivation. (**A**) Schematic overview of the experimental setup. Mice were trained for operant conditioning using the FED3 devises (VEH: n = 9; CORT: n = 10). After training completion, mice received a bottle with CORT (100 μg/ml) or VEH in their drinking solution for 10 consecutive days. On day 10 and day 17, mice were tested for effort-based reward motivation (PR1 and PR2, respectively). Peripheral blood was drawn on day 0, 10 and 17. (**B**) We observed increased CORT levels at day 10, but found no differences at baseline (day 0) of at day 17 (Effect of stress: F_1,50_ = 14.72, *P* = 0.0004). (**C**) During PR1, the number of correct nosepokes were reduced for CORT-treated mice (t = 1.8, df = 17, *P* = 0.045) whereas (**D**) no differences were found for the frequency of incorrect nosepokes (t = 1.2, df = 17, *P* = 0.24). During PR2, (**E**) correct nosepokes were similar for VEH and CORT-treated mice (t = 1.2, df = 17, *P* = 0.24), while (**F**) the number of incorrect nosepokes was increased for CORT-treated mice (t = 2.2, df = 17, *P* = 0.042). (**G**) We found that the accuracy during PR1 was similar for VEH and CORT-treated mice, but was reduced during PR2 exclusively for CORT-treated mice (Effect of time F_1,17_ = 14.9, *P* = 0.0013, effect of CORT vs. VEH on PR1: t = 0.17, *P* = 0.86 and PR2: t = 2.32, *P* = 0.027). ∗*P* < 0.05, ∗∗*P* < 0.01. Abbreviations PR: progressive ratio, CORT: corticosterone, VEH: vehicle. Statistical tests used are two-way ANOVA (**B**,**G**) and Student's t-test (**C**–**F**).

Concentrations of circulating CORT in blood plasma were elevated on day 10 of CSD, but did not differ from CTRL a week later (i.e. experimental day 17) (Fig. 5A). As CORT concentrations on day 10 correlated negatively with the frequency of correct nosepokes (Fig. 5B), and not with the frequency of incorrect nosepokes (Supplementary Fig. 2), we asked whether elevated CORT levels could contribute to reduced effort-based reward motivation. To investigate whether reduced effort-based reward motivation may be causally linked to HPA axis activation, resulting in hypercortisolemia, we included another cohort of mice and supplemented their drinking water with CORT (Fig. 6A). After 10 d of CORT treatment [100 μg/ml], CORT concentrations in the peripheral blood mimicked those seen in CSD-exposed mice and one week thereafter, CORT levels were back to baseline (Fig. 6B). Of note, 10 d of CORT treatment [100 μg/ml] did not impair concurrent food- or water intake (Supplementary Table 1). At short-term (PR1) CORT-treated mice [100 μg/ml] displayed less correct nosepokes while no effect was found for the number of incorrect nosepokes (Fig. 6C–D). At long-term (PR2), no differences were seen between vehicle- and CORT-treated mice for the number of correct nosepokes (Fig. 6E). Incorrect nosepokes were increased for CORT-treated mice (Fig. 6F), contributing to a reduced accuracy at PR2, as compared to VEH-treated mice (Fig. 6G). Lowering the CORT concentrations in the drinking water [30 μg/ml] (Supplementary Fig. 3A) resulted in a markedly lower, albeit significant, increase of CORT levels in the peripheral blood after 10 d (Supplementary Fig. 3B). In response, this subliminal dose failed to affect correct nosepokes, incorrect nosepokes, or the number of reward pellets obtained (Supplementary Figs. 3C–E).

## 4 Discussion

Caloric restriction in mice reprograms the stress pathway (Pankevich et al., 2010). Therefore, food restriction paradigms confound the interpretation of data on effort-based reward motivation and this is particularly relevant when studying the impact of stress (Peng et al., 2021; Dieterich et al., 2021). Thus, although food restriction should be considered as a stressor in its own right, it was deemed a necessity, since the animals could not be trained to exert effort to obtain food rewards. Here we show, under *ad libitum* food availability, that mice can be trained within a week. Furthermore, we found that the suppressing effects of chronic social stress on effort-based reward motivation are short-lived and linked to a temporary enhancement of corticosterone levels.

We first showed that training mice in their home cage on the FED3 boxes is swift, whereby mice are fully trained within 8 days without food restriction. In contrast, food restricted mice typically require 2–3 weeks before being trained on operant conditioning outside the animals’ home cage (Rossi and Yin, 2012; Ramsey et al., 2020). Furthermore, we demonstrated that our FED3 training protocol was robust, as operant memory was retained for at least 16 days in the reminder FR5 trials, independent of treatment (CSD- or non-stressed controls). That the FED3 training was brief, yet endured, may be connected to the mice being trained 24/7 in the relative stress-free environment of their home cage, in line with previous work (Remmelink et al., 2017). An important advantage of the setup used in our study is that food restriction could be avoided completely. In addition, the reward pellets used were low in sucrose, compared to the often used 100% sucrose pellets (Hernandez and Kelley, 2004; Malkki et al., 2010; Karlsson and Cameron, 2023). Seeing the close connections between CSD and glucose metabolism (van der Kooij et al., 2018), we opted for low sucrose containing pellets, with the aim to suppress the potential impact of pellet intake on glucose metabolism. During effortless training (free feeding, FF), mice took substantial amounts of reward pellets (125 ± 11.6), during a 24 h session. As the number of reward pellets obtained during PR remained <20, we exclude the possibility that satiety confounded effort-based reward motivation. Importantly, despite *ad libitum* food (chow) availability, mice were willing to exert effort to obtain the food pellets, as evidenced by the substantial numbers of nosepokes at high accuracy during PR.

Social defeat stress in mice has been reported to increase (non-effort based) palatable food intake and preference under *ad libitum* access (Linders et al., 2022). We found, acutely after stress, i.e. during PR1, that mice exerted less effort to obtain the highly palatable food reward pellets (Fig. 3A,B,E), emphasizing the impact of stress on the exertion of effort. Other studies also reported reduced effort based reward motivation in acute response to chronic social stress (Bergamini et al., 2016; Bergamini et al., 2018; Kúkel'ová et al., 2018; Yoshida et al., 2021). Notably, however, we simultaneously observed a stark reduction in incorrect nosepokes (Fig. 3C and D). Therefore, during the PR performed at day 10 of CSD, a stress-induced reduction in general activity may underlie an overall lower response rate, rather than a specific reduction in reinforced responses. Interestingly, in another study, mice were also continuously monitored in their home-cage (IntelliCage). Herein, a generalized reduction in nosepoke responses (both for saccharin rewards and for water) was also observed for CSD-exposed mice (Bergamini et al., 2016). Hence, these findings suggest that CSD impacts the exertion of effort. Of note, recent work involving chronic restraint stress, demonstrated a dissociation between motor activity and effort-based reward motivation (Scott et al., 2023). To conclude, we cannot exclude that the effects of CSD on PR1 comprised a *bona fide* impact on effort-based reward motivation. Regardless of the potential involvement of motor function for the impaired PR1 performance in stressed mice, we surmised that acute CSD-induced effects on PR performance are short-lived and connected to hypercortisolemia.

To start, CORT levels were increased at day 10, but not at day 17 (Fig. 5A). Moreover, on day 10, CORT concentrations correlated negatively with the number of correct nosepokes during PR (Fig. 5B). We then assessed whether the impact of CSD on effort-based reward motivation would be replicated by supplementing the drinking water with CORT. Since CORT does not dissolve well in water, it is often dissolved in ethanol or DMSO (Boersma et al., 2016; Dominguez et al., 2019). Seeing the potential confounding behavioral effects of ethanol and DMSO on motor activity (Castro et al., 1995), operant behavior (Lopez and Becker, 2014) and the interactions between stress and alcohol consumption, particularly in a home cage setting (Noori et al., 2014), we opted to use beta-cyclodextrin as dissolvent. Cyclodextrins enhance solubility of hydrophobic compounds, such as CORT, and no behavioral side-effects have been reported (Loftsson and Duchêne, 2007). CORT [100 μg/ml] administration via the drinking water mimicked the CORT levels in blood plasma of CSD-exposed mice on day 10 of treatment (Fig. 6A). We also observed that effort-based reward motivation was reduced for CORT-treated mice at PR1, but not at PR2, emulating our CSD-based findings. The potential confounding effects of CORT on food- and water intake can be excluded, since these did not differ between CORT- and VEH-treated mice (Supplementary Table 1). We noticed that accuracy for CORT-treated mice decreased, when comparing the PR performed at day 10 (PR1) and day 17 (PR2). We hypothesize that the CORT [100 μg/ml] supplied in the drinking water may have impaired animals’ long term memory for the correct poke hole (reference memory), in agreement with previous findings (Coburn-Litvak et al., 2003). Our considerably lower CORT dose [30 μg/ml] also increased blood plasma CORT concentrations at day 10 (Supplementary Fig. 3), but its effect size was below the typical response seen for CSD-exposed mice and, insufficient in affecting effort-based reward motivation.

An important finding from our study is that we failed to observe lasting negative effects from CSD on effort-based reward motivation, based on our PR findings, one week post-stress. We confirmed this observation in a separate cohort of mice (Fig. 4). Yet, we maintain that our CSD-paradigm was sufficiently stressful. First, CSD exerted a lasting impact on stress physiology, as demonstrated by the adrenal hyperplasia, three weeks after the stressor. Second, using the same CSD-paradigm, we previously reported impaired spatial memory performance as well as extended immobility in the forced swim test, one week post-CSD (van der Kooij et al., 2018). Hence, we assert that, under *ad libitum* conditions, CSD-exposed mice are not lastingly impaired in effort-based reward motivation. Interestingly, previous work showed that CSD impacted sucrose preference, as proxy for anhedonia, in non-food restricted mice acutely after stress (day 11), but not at long term (day 39) (Krishnan et al., 2007). In other work, CSD in non-food restricted rats lastingly impacted reward function, measured by intracranial self-stimulation, but this effect was only seen for stress-susceptible individuals (Der-Avakian et al., 2014). Of note, food restriction may not be the sole reason underlying a lasting impact of CSD on motivational deficits; it was shown that CSD-exposed mice, which stayed in sensory contact (co-housed with a divider) with the aggressor after CSD, maintained motivational deficits (Kúkel'ová et al., 2018).

In patients with major depression, effort-based reward motivation was found to be reduced as compared to healthy controls (Treadway et al., 2012). Chronic stress is thought to be centrally involved in the development of motivational deficits (Hershenberg et al., 2016; Salamone and Correa, 2024). However, our findings indicate that despite the initial impact of CSD on effort-based reward motivation, these motivational deficits do not persist in the long term. This resilience observed in mice, under *ad libitum* food conditions, suggests that the transient nature of stress-induced impairments may be more complex than previously thought. Our study highlights the importance of considering the temporal dynamics of stress effects and, notably, taking into account the effects of CORT. By demonstrating that mice can maintain motivation for reward even after CSD, our research underscores the robustness of adaptive responses, paving the way for future studies to explore the factors that facilitate this resilience.

## CRediT authorship contribution statement

**Danina Evertse:** Writing – review & editing, Investigation, Formal analysis. **Pilar Alves-Martinez:** Writing – review & editing, Investigation. **Giulia Treccani:** Writing – review & editing, Supervision. **Marianne B. Müller:** Supervision, Resources. **Frank J. Meye:** Writing – review & editing, Supervision, Resources. **Michael A. van der Kooij:** Writing – review & editing, Writing – original draft, Visualization, Validation, Supervision, Resources, Project administration, Methodology, Investigation, Funding acquisition, Formal analysis, Conceptualization.

## Funding

MK was funded by the 10.13039/501100001659Deutsche Forschungsgemeinschaft 10.13039/501100001659DFG, KO 5579/5–1.

## Declaration of competing interest

The authors declare no conflicts of interest.

## Data Availability

Data will be made available on request.
