# Supplementary material for: Transient impact of chronic social stress on effort-based reward motivation in non-food restricted mice: Involvement of corticosterone
Source: Neurobiol Stress. 2024 Nov 9;33:100690. doi: 10.1016/j.ynstr.2024.100690 (PMC11602574; doi:10.1016/j.ynstr.2024.100690)
Supplement: Multimedia component 1 [file mmc1.docx]

**Supplementary Information**


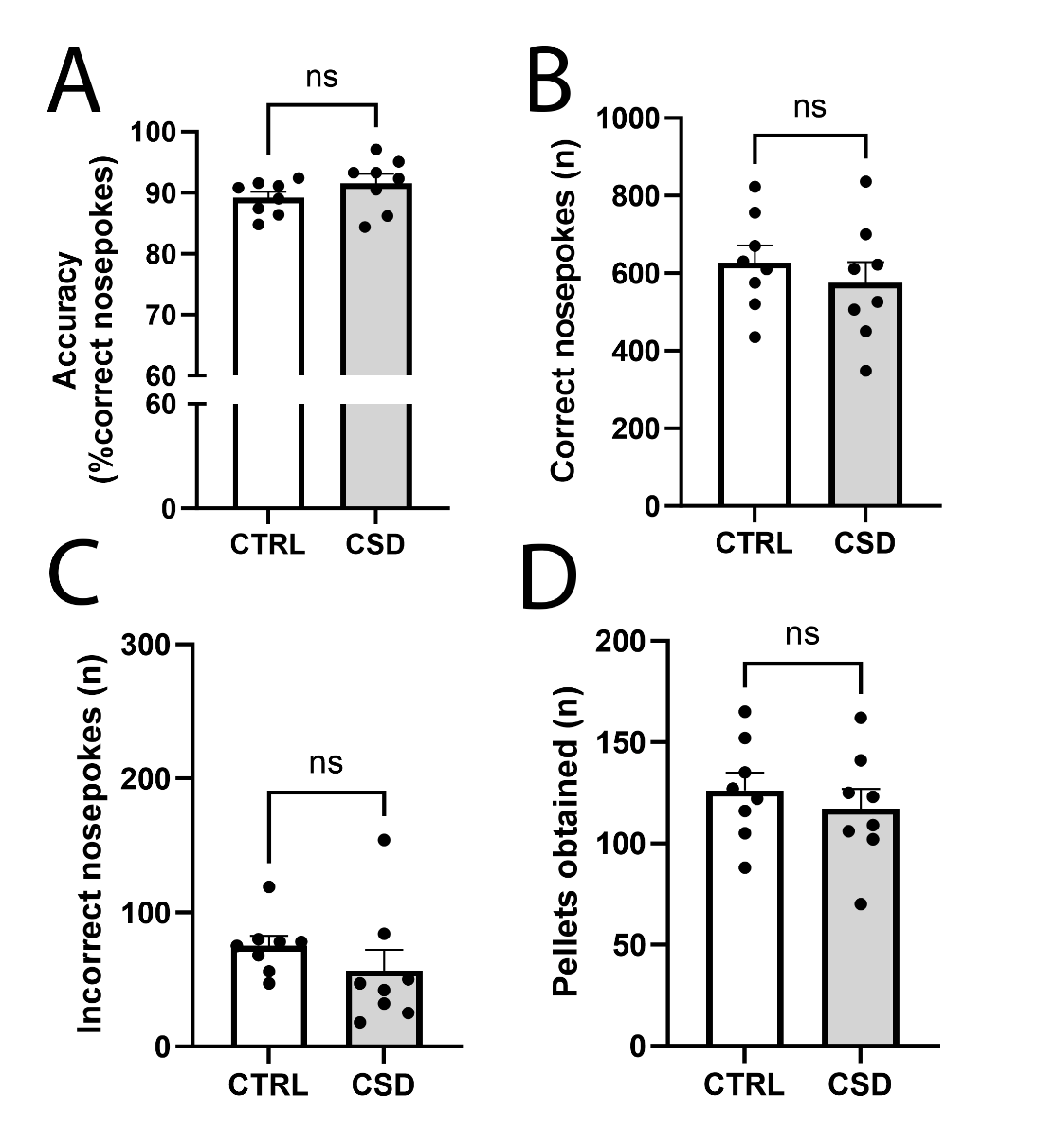


**Supplementary Fig. 1 Chronic social defeat does not affect FED3 performance during the reminder training trial six days after stress.** (**A**) Accuracy did not differ between CSD and CTRL (t= 1.29, df= 14, *P*= 0.22, n= 8/group) and nor did, (**B**) the number of correct nosepokes (t= 0.76, df= 14, *P*= 0.46, n= 8/group), (**C**) the number of incorrect nosepokes (t= 1.07, df= 14, *P*= 0.30, n= 8/group) or (**D**) the number of pellets obtained (t= 0.69, df= 14, *P*= 0.50, n= 8/group). Statistical tests used are Student’s t-test (**A**-**D**).


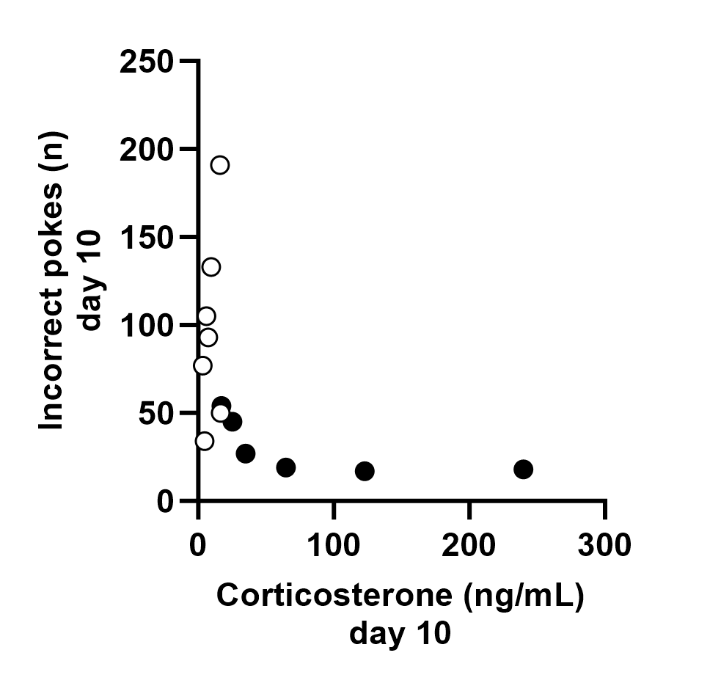


**Supplementary Fig. 2 No association between corticosterone and incorrect nose pokes during progressive ratio.** On the 10^th^ (final) day of CSD, the concentrations of corticosterone in blood plasma did not correlate with the number of incorrect nose pokes displayed during the progressive ratio (r^2^= 0.23, *P*= 0.10, n= 13).

**
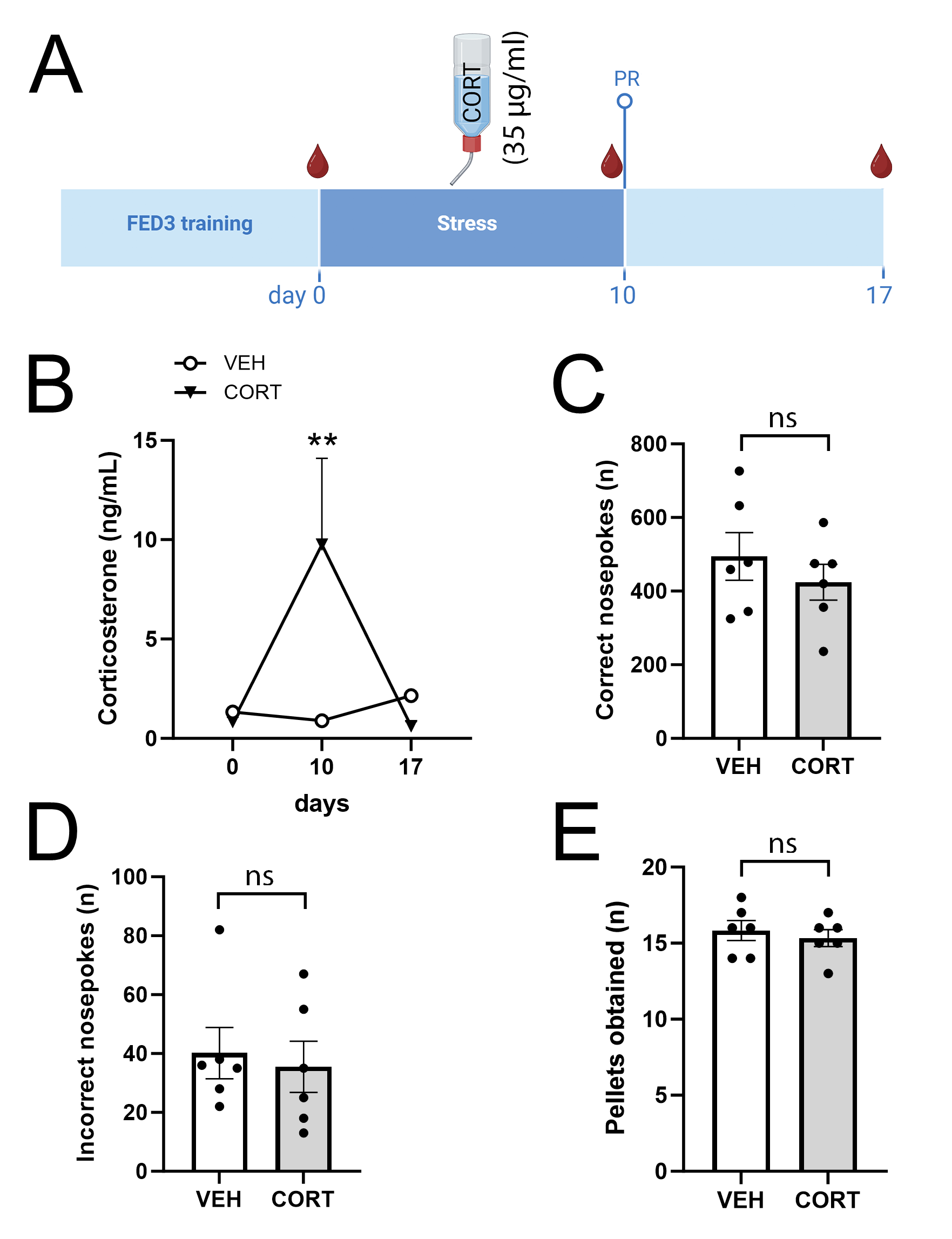
**

**Supplementary Fig. 3 A low dose of corticosterone (35 µg/ml) in the drinking water is insufficient to affect effort-based reward motivation.** (**A**) Schematic overview of the experimental setup. Mice were trained for operant conditioning using the FED3 devises (n= 6/group). After training completion, mice received a bottle with CORT (35 µg/ml) or VEH in their drinking solution for 10 consecutive days. On day 10, mice were tested for effort-based reward motivation on a PR. Peripheral blood was drawn on day 0, 10 and 17. (**B**) We observed increased CORT levels at day 10, but found no differences at baseline (day 0) of at day 17 (Effect of interaction: F_2,28_= 4.37, *P*= 0.022, day 0: t= 0.19, *P*= 1.0; day 10: t= 3.22, *P*= 0.0098; day 17: t= 0.56, *P*= 0.93). (**C**) The number of correct nosepokes did not differ between VEH and CORT (t= 0.86, df= 10, *P*= 0.41) nor did, (**D**) the number of incorrect nosepokes (t= 0.38, df= 10, *P*= 0.71) or (**E**) the number of pellets obtained (U= 14.5, *P*= 0.62). Abbreviations CORT: corticosterone; PR: progressive ratio; VEH: vehicle. Statistical tests used are two-way ANOVA (**B**), Student’s t-test (**C**-**D**) and Mann-Whitney test (**E**).

**Supplementary Table 1.** Food (chow) and water consumption during 10d of corticosterone (CORT) or vehicle (VEH) treatment, administered via the drinking water (n= 4/group).

|  | VEH | CORT | *P* |
| --- | --- | --- | --- |
| Food intake (g/d) | 3.9 ± 0.1 | 4.4 ± 0.3 | 0.11 |
| Water consumption (ml/d) | 4.6 ± 0.3 | 7.9 ± 1.8 | 0.16 |
